# Existence of entire solutions of some non-linear differential-difference equations

**DOI:** 10.1186/s13660-017-1368-1

**Published:** 2017-04-27

**Authors:** Minfeng Chen, Zongsheng Gao, Yunfei Du

**Affiliations:** LMIB and School of Mathematics and Systems Science, Beihang University, Beijing, 100191 P.R. China

**Keywords:** 39B32, 39A10, 34M05, differential-difference equation, admissible entire solution, finite order

## Abstract

In this paper, we investigate the admissible entire solutions of finite order of the differential-difference equations $(f'(z))^{2}+P^{2}(z)f^{2}(z+c)=Q(z)e^{\alpha(z)}$ and $(f'(z))^{2}+[f(z+c)-f(z)]^{2}=Q(z)e^{\alpha(z)}$, where $P(z)$, $Q(z)$ are two non-zero polynomials, $\alpha(z)$ is a polynomial and $c\in\mathbb{C}\backslash\{0\}$. In addition, we investigate the non-existence of entire solutions of finite order of the differential-difference equation $(f'(z))^{n}+P(z)f^{m}(z+c)=Q(z)$, where $P(z)$, $Q(z)$ are two non-constant polynomials, $c\in\mathbb{C}\backslash\{0\}$, *m*, *n* are positive integers and satisfy $\frac{1}{m}+\frac{1}{n}<2$ except for $m=1$, $n=2$.

## Introduction and main results

In this paper, we assume that the reader is familiar with the standard symbols and fundamental results of Nevanlinna theory [1, 2]. In addition, we denote by $S(r,f)$ any quantify satisfying $S(r,f)=o(T(r,f))$, as $r\rightarrow\infty$, outside of a possible exceptional set of finite logarithmic measure. We define the logarithmic measure of *E* to be $\operatorname{lm}(E)=\int_{E\cap(1,\infty)}\frac{dr}{r}$. A set $E\subset(1,\infty)$ is said to have finite logarithmic measure if $\operatorname{lm}(E)<\infty$. Throughout this paper, all constants are complex constants unless otherwise specified.

Nevanlinna’s value theory of meromorphic functions has been used to study the properties of entire or meromorphic solutions of differential equations and difference equations in complex plane, such as [3–5]. In [6], Montel stated the following theorem.

### Theorem A

*Let* $f(z)$, $g(z)$ *be two transcendental entire functions*. *Then if* *m* *and* *n* *are integers* ≥3, *the functional equation*

1.1 $$ f^{n}(z)+g^{n}(z)=1 $$

*cannot hold*.

However, when $n=2$ and $g(z)$ has a specific relationship with $f(z)$ in (1.1), the problem that whether we can obtain the accurate expressions of entire solutions or not is worth to be considered. Recently, many results focused on this problem that were obtained by using the Nevanlinna theory, such as [7–14].

In [10], Liu *et al.* considered Fermat type differential-difference equation and obtained the following results.

### Theorem B

*The transcendental entire solutions with finite order of*

1.2 $$ \bigl(f'(z)\bigr)^{2}+f^{2}(z+c)=1 $$

*must satisfy* $f(z)=\sin(z\pm iB)$, *where* *B* *is a constant and* $c=2k\pi$ *or* $c=2k\pi+\pi$, *k* *is an integer*.

### Theorem C

*The transcendental entire solutions with finite order of*

1.3 $$ \bigl(f'(z)\bigr)^{2}+\bigl(f(z+c)-f(z) \bigr)^{2}=1 $$

*must satisfy* $f(z)=\frac{1}{2}\sin(2z+iB)$, *where* $c=k\pi+\frac{\pi}{2}$, *k* *is an integer*, *and* *B* *is a constant*.

In [7], Chen and Gao improved Theorem B and obtained the following result.

### Theorem D

*Let* $P(z)$, $Q(z)$ *be two non*-*zero polynomials*. *If the differential*-*difference equation*

1.4 $$ \bigl(f'(z)\bigr)^{2}+P^{2}(z)f^{2}(z+c)=Q(z) $$

*admits a transcendental entire solution of finite order*, *then* $P(z)$, $Q(z)$ *reduce to constants*, *and*

$$f(z)=\frac{pe^{az+b}-qe^{-(az+b)}}{2a}, $$

*where* $a=\pm iA$, $A=\frac{(-1)^{k}k\pi}{c}$, *k* *is an integer*, *b* *is a constant and* *p*, *q*, *c* *are non*-*zero constants*.

### Remark 1.1

Equation (1.2) is a special case of (1.4). Theorem D generalized Theorem B. From Theorem D, we see that if $P(z)$ and $Q(z)$ are non-constant polynomials, then equation (1.4) has no transcendental entire solution of finite order.

In this paper, we generalize equations (1.2)-(1.4) and obtain the following results.

### Theorem 1.1

*Let* $P(z)$, $Q(z)$ *be two non*-*zero polynomials*, $c\in\mathbb{C}\backslash\{0\}$ *and* $\alpha(z)$ *be a polynomial*. *If the differential*-*difference equation*

1.5 $$ \bigl(f'(z)\bigr)^{2}+P^{2}(z)f^{2}(z+c)=Q(z)e^{\alpha(z)} $$

*admits a transcendental entire solution of finite order*, *then* $f(z)$ *must satisfy one of the following cases*:

(i) $P(z)$ *and* $Q(z)$ *reduce to constants*, *and*

$$f(z)=\frac{q_{1}e^{A_{1}z+B_{1}}}{2A_{1}}+\frac{q_{2}e^{A_{2}z+B_{2}}}{2A_{2}}, $$

*where* $A_{1}=ie^{A_{1}c}p$, $A_{2}=-ie^{A_{2}c}p$, $B_{1}$, $B_{2}$ *are constants and* $A_{1}$, $A_{2}$, $q_{1}$, $q_{2}$, *p*, *c* *are non*-*zero constants*;

(ii) $P(z)$ *reduces to a constant*, $Q(z)$ *is a polynomial with degree* 1, *and*

$$f(z)=\frac{ (a_{1}z+a_{0}-\frac{a_{1}}{A_{1}} )e^{A_{1}z+B_{1}}}{2A_{1}} +\frac{q_{2}e^{A_{2}z+B_{2}}}{2A_{2}}, \quad \frac{1}{A_{1}}=c, \frac{1}{A_{2}}\neq c, $$

*or*

$$f(z)=\frac{q_{1}e^{A_{1}z+B_{1}}}{2A_{1}} +\frac{ (b_{1}z+b_{0}-\frac{b_{1}}{A_{2}} )e^{A_{2}z+B_{2}}}{2A_{2}},\quad \frac{1}{A_{1}}\neq c, \frac{1}{A_{2}}=c, $$

*where* $A_{1}=ie^{A_{1}c}p$, $A_{2}=-ie^{A_{2}c}p$, $B_{1}$, $B_{2}$, $a_{0}$, $b_{0}$ *are constants and* $A_{1}$, $A_{2}$, $q_{1}$, $q_{2}$, $a_{1}$, $b_{1}$, *p*, *c* *are non*-*zero constants*;

(iii)

$$f(z)=B(z)e^{Az},\qquad \alpha(z)=2Az+D, $$

*where* $B(z)$ *satisfies* $[B'(z)+AB(z)]^{2}+P^{2}(z)B^{2}(z+c)e^{2Ac}=Q(z)e^{D}$, *A*, *c* *are non*-*zero constants*, *D* *is a constant*.

### Remark 1.2

In equation (1.5), when $\alpha(z)$ is a constant, then equation (1.5) reduces to equation (1.4). That is, Theorem 1.1 generalizes Theorems B and D.

### Theorem 1.2

*Let* $Q(z)$ *be a non*-*zero polynomial*, $\alpha(z)$ *be a polynomial and* $c\in\mathbb{C}\backslash\{0\}$. *If the differential*-*difference equation*

1.6 $$ \bigl(f'(z)\bigr)^{2}+\bigl[f(z+c)-f(z) \bigr]^{2}=Q(z)e^{\alpha(z)} $$

*admits a transcendental entire solution of finite order*, *then* $f(z)$ *must satisfy one of the following cases*:

(i)

$$f(z)=B(z)e^{Az}+c_{0},\qquad \alpha(z)=2Az+D, $$

*where* $B(z)$ *satisfies* $[B'(z)+AB(z)]^{2}+[B(z+c)e^{Ac}-B(z)]^{2}=Q(z)e^{D}$, *A*, *c* *are non*-*zero constants*, $c_{0}$, *D* *are constants*; *In particular*, *if* $A=\pm i$, *then*

$$f(z)=\frac{q_{1}e^{B_{1}}+q_{2}e^{B_{2}}}{-2i}e^{-iz}+c_{1}, \quad q_{1}e^{B_{1}}=q_{2}e^{B_{2}} \bigl(2e^{ic}-1\bigr), $$

*or*

$$f(z)=\frac {b_{1}e^{B_{2}}(iz+1)+i(q_{1}e^{B_{1}}+b_{0}e^{B_{2}})}{2}e^{-iz}+c_{2},\quad b_{1}(2ic+1)=-2iq_{1}e^{B_{1}-B_{2}},e^{-ic}=2, $$

*or*

$$f(z)=\frac{q_{1}e^{B_{1}}+q_{2}e^{B_{2}}}{2i}e^{iz}+c_{3},\quad q_{2}e^{B_{2}}=q_{1}e^{B_{1}} \bigl(2e^{-ic}-1\bigr), $$

*or*

$$f(z)=\frac {a_{1}e^{B_{1}}(-iz+1)-i(a_{0}e^{B_{1}}+q_{2}e^{B_{2}})}{2}e^{iz}+c_{4},\quad a_{1}(2ic-1)=-2iq_{2}e^{B_{2}-B_{1}}, e^{ic}=2, $$

*where* $c_{1}$, $c_{2}$, $c_{3}$, $c_{4}$, $B_{1}$, $B_{2}$, $a_{0}$, $b_{0}$ *are constants and* $a_{1}$, $b_{1}$, $q_{1}$, $q_{2}$, *c* *are non*-*zero constants*;

(ii)

$$f(z)=\frac{q_{1}e^{B_{1}}z}{2}+\frac{q_{2}e^{A_{2}z+B_{2}}}{2A_{2}}+c_{5}, \quad e^{A_{2}c}-1=iA_{2}, c=-i, $$

*or*

$$f(z)=\frac{q_{1}e^{A_{1}z+B_{1}}}{2A_{1}}+\frac{q_{2}e^{B_{2}}z}{2}+c_{6},\quad e^{A_{1}c}-1=-iA_{1}, c=i, $$

*where* $B_{1}$, $B_{2}$, $c_{5}$, $c_{6}$ *are constants and* $A_{1}$, $A_{2}$, $q_{1}$, $q_{2}$ *are non*-*zero constants*;

(iii)

$$f(z)=\frac{q_{1}e^{A_{1}z+B_{1}}}{2A_{1}}+\frac {q_{2}e^{A_{2}z+B_{2}}}{2A_{2}}+c_{7}, $$

*or*

$$f(z)=\frac{ (a_{1}z+a_{0}-\frac{a_{1}}{A_{1}} )e^{A_{1}z+B_{1}}}{2A_{1}} +\frac{q_{2}e^{A_{2}z+B_{2}}}{2A_{2}}+c_{8},\quad \frac{1}{A_{1}+i}=c, $$

*or*

$$f(z)=\frac{q_{1}e^{A_{1}z+B_{1}}}{2A_{1}} +\frac{ (b_{1}z+b_{0}-\frac{b_{1}}{A_{2}} )e^{A_{2}z+B_{2}}}{2A_{2}}+c_{9},\quad \frac{1}{A_{2}-i}=c, $$

*or*

$$f(z)=\frac{ (a_{1}z+a_{0}-\frac{a_{1}}{A_{1}} )e^{A_{1}z+B_{1}}}{2A_{1}} +\frac{ (b_{1}z+b_{0}-\frac{b_{1}}{A_{2}} )e^{A_{2}z+B_{2}}}{2A_{2}}+c_{10},\quad \frac{1}{A_{1}+i}=\frac{1}{A_{2}-i}=c, $$

*where* $e^{A_{1}c}-1=-iA_{1}$, $e^{A_{2}c}-1=iA_{2}$, $B_{1}$, $B_{2}$, $a_{0}$, $b_{0}$ *are constants and* $A_{1}$, $A_{2}$, $q_{1}$, $q_{2}$, $a_{1}$, $b_{1}$, *c* *are non*-*zero constants*.

### Remark 1.3

In equation (1.6), when $Q(z)$ is non-zero constant and $\alpha(z)$ is a constant, then equation (1.6) reduces to equation (1.3). That is, Theorem 1.2 generalizes Theorem C.

Fermat type functional equations were investigated by Gross [15, 16] and many others. In [17], Yang studied the Fermat type functional equation

1.7 $$ a(z)f^{n}(z)+b(z)g^{m}(z)=1, $$

where $a(z)$, $b(z)$ are small functions with respect to $f(z)$ and obtained the following result.

### Theorem E

*Let* *m*, *n* *be positive integers satisfying* $\frac{1}{m}+\frac{1}{n}<1$. *Then there are no non*-*constant entire solutions* $f(z)$ *and* $g(z)$ *that satisfy* (1.7).

When $g(z)$ has a specific relationship with $f(z)$ in (1.7), Liu et al. [10] studied the differential-difference equation

1.8 $$ \bigl(f'(z)\bigr)^{n}+f^{m}(z+c)=1, $$

and obtained the following result.

### Theorem F

*Equation* (1.8) *has no transcendental entire solutions with finite order*, *provided that* $m\neq n$, *where* *m*, *n* *are positive integers*.

Now, we generalize (1.8) and obtain the following result.

### Theorem 1.3

*Let* $P(z)$, $Q(z)$ *be two non*-*constant polynomials and* $c\in\mathbb{C}\backslash\{0\}$, *then the equation*

1.9 $$ \bigl(f'(z)\bigr)^{n}+P(z)f^{m}(z+c)=Q(z) $$

*has no transcendental entire solutions with finite order*, *provided that* $\frac{1}{m}+\frac{1}{n}<2$ *except for* $m=1$, $n=2$, *where* *m*, *n* *are positive integers*.

## Some lemmas

In order to prove our conclusions, we need some lemmas.

### Lemma 2.1

see [2, 18]

*Let* $f(z)$ *be a transcendental meromorphic solution of*

$$f^{n}P(z,f)=Q(z,f), $$

*where* $P(z,f)$ *and* $Q(z,f)$ *are polynomials in* $f(z)$ *and its derivatives with meromorphic coefficients*, *say* $\{a_{\lambda}|\lambda\in I\}$, *such that* $m(r,a_{\lambda})=S(r,f)$ *for all* $\lambda\in I$. *If the total degree of* $Q(z,f)$ *as a polynomial in* $f(z)$ *and its derivatives is* ≤*n*, *then* $m(r,P(z,f))=S(r,f)$.

### Lemma 2.2

see [19]

*Suppose that* $f_{1}(z), f_{2}(z),\ldots,f_{n}(z)$ ($n\geq2$) *are meromorphic functions and* $g_{1}(z), g_{2}(z),\ldots,g_{n}(z)$ *are entire functions satisfying the following conditions*:

1. $\sum_{j=1}^{n}f_{j}(z)e^{g_{j}(z)}\equiv0$.
2. $g_{j}(z)-g_{k}(z)$ *are not constants for* $1\leq j< k\leq n$.
3. *For* $1\leq j\leq n$, $1\leq h< k\leq n$, $T(r,f_{j}(z))=o(T(r,e^{g_{h}(z)-g_{k}(z)}))$ ($r\rightarrow\infty$, $r\notin E$).

*Then* $f_{j}(z)\equiv0$ ($j=1,\ldots, n$).

### Lemma 2.3

see [19]

*Suppose that* $f_{1}(z), f_{2}(z),\ldots,f_{n}(z)$ ($n\geq3$) *are meromorphic functions which are not constants except for* $f_{n}(z)$. *Furthermore*, *let* $\sum_{j=1}^{n} f_{j}(z)\equiv1$. *If* $f_{n}(z)\not\equiv0$ *and*

$$\sum_{j=1}^{n}N \biggl(r,\frac{1}{f_{j}(z)} \biggr) +(n-1)\sum_{j=1}^{n}\overline{N} \bigl(r,f_{j}(z)\bigr) < \bigl(\lambda+o(1)\bigr)T\bigl(r,f_{k}(z) \bigr), $$

*where* $r\in I$, $k=1,2,\ldots, n-1$ *and* $\lambda<1$, *then* $f_{n}(z)\equiv1$.

### Lemma 2.4

*Let* $Q(z)$ *be a non*-*zero polynomial and satisfy*

2.1 $$ Q(z+c)-Q(z)\equiv aQ'(z)+b, $$

*where* *a*, *c* *are non*-*zero constants*, *b* *is a constant*, *then one of the following cases holds*:

(i) *if* $b=0$ *and* $a\neq c$, *then* $Q(z)$ *reduces to a non*-*zero constant*;
(ii) *if* $b=0$ *and* $a=c$, *then* $Q(z)$ *reduces to a non*-*zero constant or* $Q(z)=a_{1}z+a_{0}$, *where* $a_{1}$ *is a non*-*zero constant*, $a_{0}$ *is a constant*;
(iii) *if* $b\neq0$ *and* $a\neq c$, *then* $Q(z)=a_{1}z+a_{0}$ *and* $b=a_{1}(c-a)$, *where* $a_{1}$ *is a non*-*zero constant*, $a_{0}$ *is a constant*;
(iv) *if* $b\neq0$ *and* $a=c$, *then* $Q(z)=a_{2}z^{2}+a_{1}z+a_{0}$ *and* $b=a_{2}c^{2}$, *where* $a_{2}$ *is a non*-*zero constant*, $a_{1}$, $a_{0}$ *are constants*.

### Proof

Denote

$$Q(z)=a_{s}z^{s}+a_{s-1}z^{s-1}+ \cdots+a_{0} \quad (a_{s}\neq0). $$

Then

$$\begin{aligned}& Q'(z)=sa_{s}z^{s-1}+(s-1)a_{s-1}z^{s-2}+ \cdots+a_{1}, \\& Q(z+c)=a_{s}(z+c)^{s}+a_{s-1}(z+c)^{s-1}+ \cdots+a_{0}, \\& Q(z+c)-Q(z)=sa_{s}cz^{s-1}+\bigl(a_{s}C_{s}^{2}c^{2}+a_{s-1}C_{s-1}^{1}c \bigr)z^{s-2}+ \cdots. \end{aligned}$$

(i) If $b=0$ and $a\neq c$, comparing the coefficients of $z^{s-1}$ on both sides of (2.1), we see that $sa_{s}c=asa_{s}$, it contradicts with $a\neq c$ and $a_{s}\neq0$.

(ii) If $b=0$, $a=c$ and $s\geq2$, comparing the coefficients of $z^{s-2}$ on both sides of (2.1), we see that $a_{s}C_{s}^{2}c^{2}+a_{s-1}C_{s-1}^{1}c=a(s-1)a_{s-1}$, then $a_{s}C_{s}^{2}c^{2}=0$, a contradiction. Thus $s\leq1$, that is, $Q(z)$ reduces to a non-zero constant or $Q(z)$ is a non-constant polynomial with degree 1.

(iii) If $b\neq0$ and $a\neq c$, $Q(z)$ is a non-zero constant, note that $b\neq0$, clearly (2.1) is a contradiction. If $s\geq2$, comparing the coefficients of $z^{s-1}$ on both sides of (2.1), we see that $sa_{s}c=asa_{s}$, a contradiction. If $s=1$, by (2.1), we see that $b=a_{1}(c-a)\neq0$.

(iv) If $b\neq0$ and $a=c$, $Q(z)$ is a non-zero constant, note that $b\neq0$, clearly (2.1) is a contradiction. If $s=1$, by (2.1), we see that $b=a_{1}(c-a)=0$, a contradiction. If $s\geq3$, comparing the coefficients of $z^{s-2}$ on both sides of (2.1), we see that $a_{s}C_{s}^{2}c^{2}+a_{s-1}C_{s-1}^{1}c=a(s-1)a_{s-1}$, then $a_{s}C_{s}^{2}c^{2}=0$, a contradiction. If $s=2$, by (2.1), we see that $b=a_{2}c^{2}\neq0$. □

### Lemma 2.5

see [3]

*Let* $\eta_{1}$, $\eta_{2}$ *be two complex numbers such that* $\eta_{1}\neq\eta_{2}$ *and let* $f(z)$ *be a finite order meromorphic function*. *Then we have*

$$m \biggl(r,\frac{f(z+\eta_{1})}{f(z+\eta_{2})} \biggr)=S(r,f). $$

## Proof of Theorem 1.1

Suppose that $f(z)$ is a transcendental entire solution of finite order of (1.5), then

3.1 $$ \bigl[f'(z)+iP(z)f(z+c)\bigr] \bigl[f'(z)-iP(z)f(z+c) \bigr]=Q(z)e^{\alpha(z)}. $$

Thus, both $f'(z)+iP(z)f(z+c)$ and $f'(z)-iP(z)f(z+c)$ are entire functions with finitely many zeros. Combining (3.1) with the Hadamard factorization theorem [19], Theorem 2.5, we assume that

$$f'(z)+iP(z)f(z+c)=Q_{1}(z)e^{\alpha_{1}(z)} $$

and

$$f'(z)-iP(z)f(z+c)=Q_{2}(z)e^{\alpha_{2}(z)}, $$

where $Q_{1}(z)$, $Q_{2}(z)$ are two non-zero polynomials, $\alpha_{1}(z)$, $\alpha_{2}(z)$ are two polynomials and cannot be constants simultaneously, otherwise $f(z)$ is a polynomial. Thus, we have

3.2 $$ f'(z)=\frac{Q_{1}(z)e^{\alpha_{1}(z)}+Q_{2}(z)e^{\alpha_{2}(z)}}{2} $$

and

3.3 $$ f(z+c)=\frac{Q_{1}(z)e^{\alpha_{1}(z)}-Q_{2}(z)e^{\alpha _{2}(z)}}{2iP(z)}. $$

Differentiating (3.3) and shifting (3.2) by replacing *z* with $z+c$, we have

3.4 $$\begin{aligned}& \frac{[Q'_{1}(z)+Q_{1}(z)\alpha_{1}'(z)]P(z)-Q_{1}(z)P'(z)}{i P^{2}(z)Q_{1}(z+c)}e^{\alpha_{1}(z)-\alpha_{1}(z+c)} \\& \quad {} -\frac{[Q'_{2}(z)+Q_{2}(z)\alpha_{2}'(z)]P(z)-Q_{2}(z)P'(z)}{i P^{2}(z)Q_{1}(z+c)}e^{\alpha_{2}(z)-\alpha_{1}(z+c)} \\& \quad {} -\frac{Q_{2}(z+c)}{Q_{1}(z+c)}e^{\alpha_{2}(z+c)-\alpha _{1}(z+c)}\equiv1. \end{aligned}$$

We deduce that $[Q'_{1}(z)+Q_{1}(z)\alpha _{1}'(z)]P(z)-Q_{1}(z)P'(z)\not\equiv0$ and $[Q'_{2}(z)+Q_{2}(z)\alpha_{2}'(z)]P(z)-Q_{2}(z)P'(z)\not\equiv0$. If $[Q'_{1}(z)+Q_{1}(z)\alpha_{1}'(z)]P(z)-Q_{1}(z)P'(z)\equiv0$, then $P(z)\equiv AQ_{1}(z)e^{\alpha_{1}(z)}$, where *A* is a non-zero constant. Note that $P(z)$, $Q_{1}(z)$ are non-zero polynomials, then $\alpha_{1}(z)$ must be a constant. Let $\alpha_{1}(z)\equiv A_{1}$. Since $\alpha_{1}(z)$ and $\alpha_{2}(z)$ cannot be constants simultaneously, thus $\alpha_{2}(z)$ cannot be a constant, then $[Q'_{2}(z)+Q_{2}(z)\alpha_{2}'(z)]P(z)-Q_{2}(z)P'(z)\not\equiv0$. Then (3.4) can be rewritten as

3.5 $$\begin{aligned}& \bigl[\bigl(Q'_{2}(z)+Q_{2}(z) \alpha_{2}'(z)\bigr)P(z)-Q_{2}(z)P'(z) \bigr]e^{\alpha_{2}(z)} +i P^{2}(z)Q_{2}(z+c)e^{\alpha_{2}(z+c)} \\& \quad {}+i P^{2}(z)Q_{1}(z+c)e^{A_{1}}=0. \end{aligned}$$

If $\deg\alpha_{2}(z)\geq2$, then $\deg\alpha_{2}(z)=\deg\alpha_{2}(z+c)\geq2$, $\deg(\alpha_{2}(z+c)-\alpha_{2}(z))\geq1$, and $e^{\alpha_{2}(z)}$, $e^{\alpha_{2}(z+c)}$, $e^{\alpha_{2}(z+c)-\alpha_{2}(z)}$ are of regular growth, by Lemma 2.2, we have

$$\bigl[Q'_{2}(z)+Q_{2}(z)\alpha_{2}'(z) \bigr]P(z)-Q_{2}(z)P'(z) \equiv i P^{2}(z)Q_{1}(z+c) \equiv i P^{2}(z)Q_{2}(z+c)\equiv0, $$

a contradiction. Thus $\deg\alpha_{2}(z)\leq1$, note that $\alpha_{2}(z)$ cannot be a constant, then $\alpha_{2}(z)=A_{2}z+B_{2}$, where $A_{2}$ is a non-zero constant. Rewriting (3.5) as

3.6 $$ H(z)e^{A_{2}z}\equiv-i P^{2}(z)Q_{1}(z+c)e^{A_{1}}, $$

where $H(z)=i P^{2}(z)Q_{2}(z+c)e^{A_{2}c+B_{2}}+ [(Q'_{2}(z)+Q_{2}(z)\alpha_{2}'(z))P(z)-Q_{2}(z)P'(z)]e^{B_{2}}$. If $H(z)\equiv0$, since $i P^{2}(z)Q_{1}(z+c)e^{A_{1}}\not\equiv0$, clearly (3.6) is a contradiction. If $H(z)\not\equiv0$, we can see that the left side of (3.6) is a transcendental entire function, and the right side of (3.6) is a non-zero polynomial, a contradiction.

Similarly, we can prove that $[Q'_{2}(z)+Q_{2}(z)\alpha_{2}'(z)]P(z)-Q_{2}(z)P'(z)\not\equiv0$.

Thus, $[Q'_{1}(z)+Q_{1}(z)\alpha_{1}'(z)]P(z)-Q_{1}(z)P'(z)\not \equiv0$ and $[Q'_{2}(z)+Q_{2}(z)\alpha_{2}'(z)]P(z)-Q_{2}(z)P'(z)\not\equiv0$, by (3.4) and Lemma 2.3, we see that if any two of $e^{\alpha_{1}(z)-\alpha_{1}(z+c)}$, $e^{\alpha_{2}(z)-\alpha_{1}(z+c)}$ and $e^{\alpha_{2}(z+c)-\alpha_{1}(z+c)}$ are not constants, then the third term must be constant. If any two of them are constants, then the third term also must be constant. In what follows, we discuss four cases: Case 1, $e^{\alpha_{1}(z)-\alpha_{1}(z+c)}$ and $e^{\alpha _{2}(z)-\alpha_{1}(z+c)}$ are not constants; Case 2, $e^{\alpha_{1}(z)-\alpha_{1}(z+c)}$ and $e^{\alpha_{2}(z+c)-\alpha_{1}(z+c)}$ are not constants; Case 3, $e^{\alpha_{2}(z)-\alpha_{1}(z+c)}$ and $e^{\alpha_{2}(z+c)-\alpha_{1}(z+c)}$ are not constants; Case 4, $e^{\alpha_{1}(z)-\alpha_{1}(z+c)}$, $e^{\alpha_{2}(z)-\alpha _{1}(z+c)}$ and $e^{\alpha_{2}(z+c)-\alpha_{1}(z+c)}$ are all constants.

*Case* 1, $e^{\alpha_{1}(z)-\alpha_{1}(z+c)}$ and $e^{\alpha_{2}(z)-\alpha_{1}(z+c)}$ are not constants, by (3.4) and Lemma 2.3, we have

3.7 $$ -\frac{Q_{2}(z+c)}{Q_{1}(z+c)}e^{\alpha_{2}(z+c)-\alpha_{1}(z+c)}\equiv 1, $$

which implies that $\alpha_{2}(z+c)-\alpha_{1}(z+c)$ is a constant, and

3.8 $$ \frac{[Q'_{1}(z)+Q_{1}(z)\alpha_{1}'(z)]P(z)-Q_{1}(z)P'(z)}{ [Q'_{2}(z)+Q_{2}(z)\alpha_{2}'(z)]P(z)-Q_{2}(z)P'(z)}e^{\alpha _{1}(z)-\alpha_{2}(z)}\equiv1, $$

which implies that $\alpha_{1}(z)-\alpha_{2}(z)$ is a constant.

Denote $e^{\alpha_{2}(z+c)-\alpha_{1}(z+c)}=e^{\alpha _{2}(z)-\alpha_{1}(z)}=k$ (≠0), by (3.7), we get $Q_{1}(z)=-kQ_{2}(z)$, substituting it into (3.8) yields

$$2\bigl[P'(z)Q_{2}(z)-P(z)Q_{2}'(z) \bigr]\equiv P(z)Q_{2}(z)\bigl[\alpha_{1}'(z)+ \alpha _{2}'(z)\bigr]. $$

Since $P(z)$ and $Q_{2}(z)$ are non-zero polynomials, $\alpha_{1}(z)$ and $\alpha_{2}(z)$ are polynomials, from the above identity, we get $\alpha_{1}'(z)+\alpha_{2}'(z)\equiv0$, that is, $\alpha_{1}(z)+\alpha_{2}(z)$ is a constant. Note that $\alpha_{1}(z)-\alpha_{2}(z)$ is a constant, then both $\alpha_{1}(z)$ and $\alpha_{2}(z)$ are constants, a contradiction.

*Case* 2, $e^{\alpha_{1}(z)-\alpha_{1}(z+c)}$ and $e^{\alpha_{2}(z+c)-\alpha_{1}(z+c)}$ are not constants, by (3.4) and Lemma 2.3, we have

$$-\frac{[Q'_{2}(z)+Q_{2}(z)\alpha_{2}'(z)]P(z)-Q_{2}(z)P'(z)}{i P^{2}(z)Q_{1}(z+c)}e^{\alpha_{2}(z)-\alpha_{1}(z+c)}\equiv1, $$

which implies that $\alpha_{2}(z)-\alpha_{1}(z+c)$ is a constant, then $\alpha_{2}(z+c)-\alpha_{1}(z+2c)$ is also a constant, and

$$\frac{[Q'_{1}(z)+Q_{1}(z)\alpha_{1}'(z)]P(z)-Q_{1}(z)P'(z)}{i P^{2}(z)Q_{2}(z+c)}e^{\alpha_{1}(z)-\alpha_{2}(z+c)}\equiv1, $$

which implies that $\alpha_{1}(z)-\alpha_{2}(z+c)$ is a constant.

By $\alpha_{1}(z)-\alpha_{1}(z+2c)=[\alpha_{1}(z)-\alpha _{2}(z+c)]+[\alpha_{2}(z+c)-\alpha_{1}(z+2c)]$, we see that $\alpha_{1}(z)-\alpha_{1}(z+2c)$ is a constant, then $\alpha_{1}(z)$ is a constant or a polynomial with degree 1, which implies that $\alpha_{1}(z)-\alpha_{1}(z+c)$ is also a constant, a contradiction.

*Case* 3, $e^{\alpha_{2}(z)-\alpha_{1}(z+c)}$ and $e^{\alpha_{2}(z+c)-\alpha_{1}(z+c)}$ are not constants, by (3.4) and Lemma 2.3, we have

3.9 $$ \frac{[Q'_{1}(z)+Q_{1}(z)\alpha_{1}'(z)]P(z)-Q_{1}(z)P'(z)}{ i P^{2}(z)Q_{1}(z+c)}e^{\alpha_{1}(z)-\alpha_{1}(z+c)}\equiv1, $$

which implies that $\alpha_{1}(z)-\alpha_{1}(z+c)$ is a constant, note that $[Q'_{1}(z)+Q_{1}(z)\alpha_{1}'(z)]P(z)-Q_{1}(z)P'(z)\not \equiv0$, then $\alpha_{1}(z)$ cannot be a constant, therefore, $\alpha_{1}(z)$ can only be a polynomial with degree 1. Denote $\alpha_{1}(z)=A_{1}z+B_{1}$, where $A_{1}$ is a non-zero constant, and $B_{1}$ is a constant. Rewriting (3.9) as

3.10 $$ A_{1}P(z)Q_{1}(z)+P(z)Q_{1}'(z)-P'(z)Q_{1}(z) \equiv ie^{A_{1}c}P^{2}(z)Q_{1}(z+c), $$

$P(z)$ must be a constant, denoted $P(z)\equiv p$ (≠0). Then (3.10) can be rewritten as

$$Q_{1}(z+c)-Q_{1}(z)\equiv\frac{1}{A_{1}}Q_{1}'(z) \quad \mbox{and}\quad A_{1}=ie^{A_{1}c}p. $$

By Lemma 2.4, we have (i) if $\frac{1}{A_{1}}\neq c$, then $Q_{1}(z)\equiv q_{1}$ (constant); (ii) if $\frac{1}{A_{1}}=c$, then $Q_{1}(z)\equiv q_{1}$ (constant) or $Q_{1}(z)=a_{1}z+a_{0}$, where $a_{1}$ is a non-zero constant and $a_{0}$ is a constant.

By (3.4) and (3.9), we have

3.11 $$ -\frac{[Q'_{2}(z)+Q_{2}(z)\alpha_{2}'(z)]P(z)-Q_{2}(z)P'(z)}{ i P^{2}(z)Q_{2}(z+c)}e^{\alpha_{2}(z)-\alpha_{2}(z+c)}\equiv1, $$

which implies that $\alpha_{2}(z)-\alpha_{2}(z+c)$ is a constant, note that $[Q'_{2}(z)+Q_{2}(z)\alpha_{2}'(z)]P(z)-Q_{2}(z)P'(z)\not \equiv0$, so $\alpha_{2}(z)$ cannot be a constant, then $\alpha_{2}(z)$ can only be a polynomial with degree 1, denote $\alpha_{2}(z)=A_{2}z+B_{2}$, where $A_{2}$ is a non-zero constant and $B_{2}$ is a constant. Note that $P(z)\equiv p$ (≠0), then (3.11) can be rewritten as

$$Q_{2}(z+c)-Q_{2}(z)\equiv\frac{1}{A_{2}}Q_{2}'(z) \quad \mbox{and}\quad A_{2}=-ie^{A_{2}c}p. $$

By Lemma 2.4, we have (i) if $\frac{1}{A_{2}}\neq c$, then $Q_{2}(z)\equiv q_{2}$ (constant); (ii) if $\frac{1}{A_{2}}=c$, then $Q_{2}(z)\equiv q_{2}$ (constant) or $Q_{2}(z)=b_{1}z+b_{0}$, where $b_{1}$ is a non-zero constant and $b_{0}$ is a constant.

Note that $A_{1}=ie^{A_{1}c}p$ and $A_{2}=-ie^{A_{2}c}p$, we see that $A_{1}\neq A_{2}$, that is, $\frac{1}{A_{1}}\neq\frac{1}{A_{2}}$. In what follows, we discuss three subcases: Subcase 3.1, $\frac{1}{A_{1}}\neq c$ and $\frac{1}{A_{2}}\neq c$; Subcase 3.2, $\frac{1}{A_{1}}=c$ and $\frac{1}{A_{2}}\neq c$; Subcase 3.3, $\frac{1}{A_{1}}\neq c$ and $\frac{1}{A_{2}}=c$.

*Subcase* 3.1, $\frac{1}{A_{1}}\neq c$ and $\frac{1}{A_{2}}\neq c$, then $Q_{1}(z)\equiv q_{1}$ and $Q_{2}(z)\equiv q_{2}$, by (1.5), (3.2) and (3.3), we obtain

$$f'(z)=\frac{q_{1}e^{A_{1}z+B_{1}}+q_{2}e^{A_{2}z+B_{2}}}{2} $$

and

$$f(z)=\frac{q_{1}e^{A_{1}z+B_{1}}}{2A_{1}}+\frac{q_{2}e^{A_{2}z+B_{2}}}{2A_{2}}. $$

*Subcase* 3.2, $\frac{1}{A_{1}}=c$ and $\frac{1}{A_{2}}\neq c$, then $Q_{1}(z)\equiv q_{1}$ and $Q_{2}(z)\equiv q_{2}$ or $Q_{1}(z)=a_{1}z+a_{0}$ and $Q_{2}(z)\equiv q_{2}$. If $Q_{1}(z)\equiv q_{1}$ and $Q_{2}(z)\equiv q_{2}$, the same as Subcase 3.1. If $Q_{1}(z)=a_{1}z+a_{0}$ and $Q_{2}(z)\equiv q_{2}$, by (1.5), (3.2) and (3.3), we obtain

$$f'(z)=\frac{(a_{1}z+a_{0})e^{A_{1}z+B_{1}}+q_{2}e^{A_{2}z+B_{2}}}{2} $$

and

$$f(z)=\frac{ (a_{1}z+a_{0}-\frac{a_{1}}{A_{1}} )e^{A_{1}z+B_{1}}}{2A_{1}} +\frac{q_{2}e^{A_{2}z+B_{2}}}{2A_{2}}. $$

*Subcase* 3.3, $\frac{1}{A_{1}}\neq c$ and $\frac{1}{A_{2}}=c$, then $Q_{1}(z)\equiv q_{1}$ and $Q_{2}(z)\equiv q_{2}$ or $Q_{1}(z)\equiv q_{1}$ and $Q_{2}(z)=b_{1}z+b_{0}$. If $Q_{1}(z)\equiv q_{1}$ and $Q_{2}(z)\equiv q_{2}$, the same as Subcase 3.1. If $Q_{1}(z)\equiv q_{1}$ and $Q_{2}(z)=b_{1}z+b_{0}$, by (1.5), (3.2) and (3.3), we obtain

$$f'(z)=\frac{q_{1}e^{A_{1}z+B_{1}}+(b_{1}z+b_{0})e^{A_{2}z+B_{2}}}{2} $$

and

$$f(z)=\frac{q_{1}e^{A_{1}z+B_{1}}}{2A_{1}} +\frac{ (b_{1}z+b_{0}-\frac{b_{1}}{A_{2}} )e^{A_{2}z+B_{2}}}{2A_{2}}. $$

*Case* 4, $e^{\alpha_{1}(z)-\alpha_{1}(z+c)}$, $e^{\alpha_{2}(z)-\alpha_{1}(z+c)}$ and $e^{\alpha_{2}(z+c)-\alpha_{1}(z+c)}$ are all constants, that is, $\alpha_{1}(z)-\alpha_{1}(z+c)$, $\alpha_{2}(z)-\alpha_{1}(z+c)$ and $\alpha_{2}(z+c)-\alpha_{1}(z+c)$ are all constants. Note that $\alpha_{1}(z)$ and $\alpha_{2}(z)$ are not constants simultaneously, then $\alpha_{1}(z)=Az+B_{1}$, $\alpha_{2}(z)=Az+B_{2}$ and $\alpha(z)=2Az+D$, where *A* is non-zero constant and $B_{1}$, $B_{2}$, *D* ($=B_{1}+B_{2}$) are constants. Therefore, by (1.5), (3.2) and (3.3), we have $f(z)=B(z)e^{Az}$, where $B(z)$ satisfies $[B'(z)+AB(z)]^{2}+P^{2}(z)B^{2}(z+c)e^{2Ac}=Q(z)e^{D}$.

This completes the proof of Theorem 1.1.

## Proof of Theorem 1.2

As in the beginning of the proof of Theorem 1.1, from (1.6), we have

4.1 $$ f'(z)=\frac{Q_{1}(z)e^{\alpha_{1}(z)}+Q_{2}(z)e^{\alpha_{2}(z)}}{2} $$

and

4.2 $$ f(z+c)-f(z)=\frac{Q_{1}(z)e^{\alpha_{1}(z)}-Q_{2}(z)e^{\alpha _{2}(z)}}{2i}. $$

where $Q_{1}(z)$, $Q_{2}(z)$ are two non-zero polynomials, $\alpha_{1}(z)$, $\alpha_{2}(z)$ are two polynomials and cannot be constants simultaneously, otherwise $f(z)$ is a polynomial. Differentiating (4.2), shifting (4.1) by replacing *z* with $z+c$ and combining (4.1), we have

4.3 $$\begin{aligned}& \frac{Q'_{1}(z)+Q_{1}(z)\alpha_{1}'(z)+iQ_{1}(z)}{iQ_{1}(z+c)}e^{\alpha _{1}(z)-\alpha_{1}(z+c)} \\& \quad {}-\frac{Q'_{2}(z)+Q_{2}(z)\alpha _{2}'(z)-iQ_{2}(z)}{iQ_{1}(z+c)}e^{\alpha_{2}(z)-\alpha_{1}(z+c)} -\frac{Q_{2}(z+c)}{Q_{1}(z+c)}e^{\alpha_{2}(z+c)-\alpha_{1}(z+c)} \equiv1. \end{aligned}$$

If $Q'_{1}(z)+Q_{1}(z)\alpha_{1}'(z)+iQ_{1}(z)\equiv0$, that is, $-Q'_{1}(z)=(\alpha_{1}'(z)+i)Q_{1}(z)$, then we have $\alpha_{1}'(z)+i\equiv0$ and $Q_{1}(z)\equiv q_{1}$ (constant). Thus $\alpha_{1}(z)=-iz+B_{1}$, where $B_{1}$ is a constant, substitute it into (4.3) yields

4.4 $$ \bigl[Q'_{2}(z)+Q_{2}(z) \bigl( \alpha_{2}'(z)-i\bigr)\bigr]e^{\alpha_{2}(z)} +iQ_{2}(z+c)e^{\alpha_{2}(z+c)}+iQ_{1}(z+c)e^{\alpha_{1}(z+c)} \equiv 0. $$

Suppose $\deg\alpha_{2}(z)\geq2$. Clearly, $Q'_{2}(z)+Q_{2}(z)(\alpha_{2}'(z)-i)\not\equiv0$. According to $\deg\alpha_{2}(z)=\deg\alpha_{2}(z+c)\geq2$, $\deg\alpha_{1}(z+c)=1$, $\deg(\alpha_{2}(z+c)-\alpha_{1}(z+c))=\deg(\alpha_{2}(z)-\alpha _{1}(z+c))\geq2$ and $\deg(\alpha_{2}(z+c)-\alpha_{2}(z))\geq1$, and $e^{\alpha_{2}(z)}$, $e^{\alpha_{2}(z+c)}$, $e^{\alpha_{1}(z+c)}$, $e^{\alpha_{2}(z+c)-\alpha_{1}(z+c)}$, $e^{\alpha_{2}(z)-\alpha_{1}(z+c)}$, $e^{\alpha_{2}(z+c)-\alpha_{2}(z)}$ are of regular growth, by Lemma 2.2, we have

$$Q'_{2}(z)+Q_{2}(z) \bigl(\alpha_{2}'(z)-i \bigr)\equiv iQ_{1}(z+c)\equiv iQ_{2}(z+c)\equiv0, $$

a contradiction. Thus $\deg\alpha_{2}(z)\leq1$, that is, $\alpha_{2}(z)=B_{2}$ (constant) or $\alpha_{2}(z)=A_{2}z+B_{2}$, where $A_{2}$ is a non-zero constant. If $\alpha_{2}(z)\equiv B_{2}$, by (4.4), we have $e^{\alpha_{1}(z+c)}\equiv-\frac {e^{B_{2}}[Q'_{2}(z)-iQ_{2}(z)+iQ_{2}(z+c)]}{iQ_{1}(z+c)}$, the left side of this identity is a transcendental entire function, and the right side of this identity is a rational function, a contradiction. Hence, $\alpha_{2}(z)=A_{2}z+B_{2}$. Rewriting (4.4) as

$$-iq_{1}e^{(-i-A_{2})z-ic+B_{1}-B_{2}}\equiv Q'_{2}(z)+(A_{2}-i)Q_{2}(z)+ie^{A_{2}c}Q_{2}(z+c). $$

If $-i-A_{2}\neq0$, clearly the above identity is a contradiction. Then $A_{2}=-i$. The above identity can be rewritten as

4.5 $$ 2iQ_{2}(z)-Q'_{2}(z)-iq_{1}e^{-ic+B_{1}-B_{2}} \equiv ie^{-ic}Q_{2}(z+c). $$

If $Q_{2}(z)\equiv q_{2}$ (constant), by (4.5), we get $q_{1}e^{B_{1}}=q_{2}e^{B_{2}}(2e^{ic}-1)$. By (1.6), (4.1) and (4.2), we have

$$f'(z)=\frac{q_{1}e^{B_{1}}+q_{2}e^{B_{2}}}{2}e^{-iz} $$

and

$$f(z)=\frac{q_{1}e^{B_{1}}+q_{2}e^{B_{2}}}{-2i}e^{-iz}+c_{1}. $$

If $Q_{2}(z)$ is a non-constant polynomial, by (4.5), we obtain

$$2i\bigl[Q_{2}(z+c)-Q_{2}(z)\bigr]=-Q'_{2}(z)-iq_{1}e^{-ic+B_{1}-B_{2}}, \quad 2i=ie^{-ic}. $$

Note that $-iq_{1}e^{-ic+B_{1}-B_{2}}\neq0$ and $-\frac{1}{2i}\neq c$, by Lemma 2.4, we see that $Q_{2}(z)=b_{1}z+b_{0}$, where $b_{1}$ is a non-zero constant and $b_{0}$ is a constant. From (4.5), we get $b_{1}(2ic+1)=-2iq_{1}e^{B_{1}-B_{2}}$, by (1.6), (4.1) and (4.2), we have

$$f'(z)=\frac{(b_{1}z+b_{0})e^{B_{2}}+q_{1}e^{B_{1}}}{2}e^{-iz} $$

and

$$f(z)=\frac {b_{1}e^{B_{2}}(iz+1)+i(q_{1}e^{B_{1}}+b_{0}e^{B_{2}})}{2}e^{-iz}+c_{2}. $$

Similarly, if $Q'_{2}(z)+Q_{2}(z)\alpha_{2}'(z)-iQ_{2}(z)\equiv0$, then we have

$$f(z)=\frac{q_{1}e^{B_{1}}+q_{2}e^{B_{2}}}{2i}e^{iz}+c_{3},\quad q_{2}e^{B_{2}}=q_{1}e^{B_{1}} \bigl(2e^{-ic}-1\bigr), $$

or

$$f(z)=\frac {a_{1}e^{B_{1}}(-iz+1)-i(a_{0}e^{B_{1}}+q_{2}e^{B_{2}})}{2}e^{iz}+c_{4}, \quad a_{1}(2ic-1)=-2iq_{2}e^{B_{2}-B_{1}}, e^{ic}=2, $$

where $c_{3}$, $c_{4}$, $B_{1}$, $B_{2}$, $a_{0}$ are constants and $a_{1}$, $q_{1}$, $q_{2}$ are non-zero constants.

According to the above proof, we can see that $Q'_{1}(z)+Q_{1}(z)\alpha_{1}'(z)+iQ_{1}(z)\equiv0$ and $Q'_{2}(z)+Q_{2}(z)\alpha_{2}'(z)-iQ_{2}(z)\equiv0$ cannot be valid simultaneously. In what follows, we assume that $Q'_{1}(z)+Q_{1}(z)\alpha_{1}'(z)+iQ_{1}(z)\not\equiv0$ and $Q'_{2}(z)+Q_{2}(z)\alpha_{2}'(z)-iQ_{2}(z)\not\equiv0$. By (4.3) and Lemma 2.3, we see that if any two of $e^{\alpha_{1}(z)-\alpha_{1}(z+c)}$, $e^{\alpha_{2}(z)-\alpha_{1}(z+c)}$ and $e^{\alpha_{2}(z+c)-\alpha_{1}(z+c)}$ are not constants, then the third term must be constant. If any two of them are constants, then the third term also must be constant. In the following, we discuss four cases: Case 1, $e^{\alpha_{1}(z)-\alpha_{1}(z+c)}$ and $e^{\alpha _{2}(z)-\alpha_{1}(z+c)}$ are not constants; Case 2, $e^{\alpha_{1}(z)-\alpha_{1}(z+c)}$ and $e^{\alpha_{2}(z+c)-\alpha_{1}(z+c)}$ are not constants; Case 3, $e^{\alpha_{2}(z)-\alpha_{1}(z+c)}$ and $e^{\alpha_{2}(z+c)-\alpha_{1}(z+c)}$ are not constants; Case 4, $e^{\alpha_{1}(z)-\alpha_{1}(z+c)}$, $e^{\alpha_{2}(z)-\alpha _{1}(z+c)}$ and $e^{\alpha_{2}(z+c)-\alpha_{1}(z+c)}$ are all constants.

*Case* 1 and *Case* 2, similarly to the proof of Case 1 and Case 2 of Theorem 1.1, we can obtain a contradiction.

*Case* 3, $e^{\alpha_{2}(z)-\alpha_{1}(z+c)}$ and $e^{\alpha_{2}(z+c)-\alpha_{1}(z+c)}$ are not constants, by (4.3) and Lemma 2.3, we have

4.6 $$ \frac{Q'_{1}(z)+Q_{1}(z)\alpha_{1}'(z)+iQ_{1}(z)}{iQ_{1}(z+c)}e^{\alpha _{1}(z)-\alpha_{1}(z+c)}\equiv1, $$

which implies that $\alpha_{1}(z)-\alpha_{1}(z+c)$ is a constant, then $\alpha_{1}(z)=A_{1}z+B_{1}$ or $\alpha_{1}(z)\equiv B_{1}$, where $A_{1}$ is a non-zero constant and $B_{1}$ is a constant. By (4.3) and (4.7), we also have

4.7 $$ -\frac{Q'_{2}(z)+Q_{2}(z)\alpha_{2}'(z)-iQ_{2}(z)}{iQ_{2}(z+c)}e^{\alpha _{2}(z)-\alpha_{2}(z+c)}\equiv1, $$

which implies that $\alpha_{2}(z)-\alpha_{2}(z+c)$ is a constant, then $\alpha_{2}(z)=A_{2}z+B_{2}$ or $\alpha_{2}(z)\equiv B_{2}$, where $A_{2}$ is a non-zero constant and $B_{2}$ is a constant. Note that $\alpha_{1}(z)$ and $\alpha_{2}(z)$ cannot be constants simultaneously, In what follows, we discuss three subcases: Subcase 3.1, $\alpha_{1}(z)\equiv B_{1}$ and $\alpha_{2}(z)=A_{2}z+B_{2}$; Subcase 3.2, $\alpha_{1}(z)=A_{1}z+B_{1}$ and $\alpha_{2}(z)\equiv B_{2}$; Subcase 3.3, $\alpha_{1}(z)=A_{1}z+B_{1}$ and $\alpha_{2}(z)=A_{2}z+B_{2}$.

*Subcase* 3.1, $\alpha_{1}(z)\equiv B_{1}$ and $\alpha_{2}(z)=A_{2}z+B_{2}$, by (4.6), we have

$$Q_{1}(z+c)-Q_{1}(z)\equiv-iQ_{1}'(z). $$

By Lemma 2.4, we see that if $c\neq-i$, then $Q_{1}(z)\equiv q_{1}$ (constant); If $c=-i$, then $Q_{1}(z)\equiv q_{1}$ (constant) or $Q_{1}(z)=a_{1}z+a_{0}$, where $a_{1}$ is a non-zero constant and $a_{0}$ is a constant.

By (4.7), we have

$$Q_{2}(z+c)-Q_{2}(z)\equiv\frac{1}{A_{2}-i}Q_{2}'(z) \quad \mbox{and}\quad A_{2}-i=-ie^{A_{2}c}. $$

By Lemma 2.4, we see that if $\frac{1}{A_{2}-i}\neq c$, then $Q_{2}(z)=q_{2}$ (constant); If $\frac{1}{A_{2}-i}=c$, then $Q_{2}(z)\equiv q_{2}$ (constant) or $Q_{2}(z)=b_{1}z+b_{0}$, where $b_{1}$ is a non-zero constant and $b_{0}$ is a constant.

If $Q_{1}(z)\equiv q_{1}$ and $Q_{2}(z)\equiv q_{2}$, by (1.6), (4.1) and (4.2), we get $c=-i$. Note that $A_{2}-i=-ie^{A_{2}c}$, then $\frac{1}{A_{2}-i}\neq-i$. Therefore, we only need to consider three subcases: Subcase 3.1.1, $\frac{1}{A_{2}-i}\neq c$ ($=-i$), $Q_{1}(z)\equiv q_{1}$ and $Q_{2}(z)\equiv q_{2}$; Subcase 3.1.2, $\frac{1}{A_{2}-i}\neq c$ ($=-i$), $Q_{1}(z)=a_{1}z+a_{0}$ and $Q_{2}(z)\equiv q_{2}$; Subcase 3.1.3, $\frac{1}{A_{2}-i}=c$ ($\neq-i$), $Q_{1}(z)\equiv q_{1}$ and $Q_{2}(z)=b_{1}z+b_{0}$.

*Subcase* 3.1.1, $\frac{1}{A_{2}-i}\neq c$ ($=-i$), $Q_{1}(z)\equiv q_{1}$ and $Q_{2}(z)\equiv q_{2}$, by (1.6), (4.1) and (4.2), we get

$$f'(z)=\frac{q_{1}e^{B_{1}}+q_{2}e^{A_{2}z+B_{2}}}{2} $$

and

$$f(z)=\frac{q_{1}e^{B_{1}}z}{2}+\frac{q_{2}e^{A_{2}z+B_{2}}}{2A_{2}}+c_{5}. $$

*Subcase* 3.1.2, $\frac{1}{A_{2}-i}\neq c$ ($=-i$), $Q_{1}(z)=a_{1}z+a_{0}$ and $Q_{2}(z)\equiv q_{2}$, by (1.6), (4.1) and (4.2), we have

$$\begin{aligned}& f'(z)=\frac{(a_{1}z+a_{0})e^{B_{1}}+q_{2}e^{A_{2}z+B_{2}}}{2}, \\& f(z)=\frac{a_{1}e^{B_{1}}z^{2}}{4}+\frac{a_{0}e^{B_{1}}z}{2} +\frac{q_{2}e^{A_{2}z+B_{2}}}{2A_{2}}+c_{5}', \end{aligned}$$

and

$$\begin{aligned} f(z+c)-f(z) =& \frac{(a_{1}z+a_{0})e^{B_{1}}-q_{2}e^{A_{2}z+B_{2}}}{2i} -\frac{a_{1}e^{B_{1}}}{4} \\ =& \frac{(a_{1}z+a_{0})e^{B_{1}}-q_{2}e^{A_{2}z+B_{2}}}{2i}, \end{aligned}$$

then $a_{1}$ must be zero, a contradiction.

*Subcase* 3.1.3, $\frac{1}{A_{2}-i}=c$ ($\neq-i$), $Q_{1}(z)\equiv q_{1}$ and $Q_{2}(z)=b_{1}z+b_{0}$, by (1.6), (4.1) and (4.2), we have

$$\begin{aligned}& f'(z)=\frac{q_{1}e^{B_{1}}+(b_{1}z+b_{0})e^{A_{2}z+B_{2}}}{2}, \\& f(z)=\frac{q_{1}e^{B_{1}}z}{2} +\frac{ (b_{1}z+b_{0}-\frac{b_{1}}{A_{2}} )e^{A_{2}z+B_{2}}}{2A_{2}}+c_{5}'', \end{aligned}$$

and

$$\begin{aligned} f(z+c)-f(z) =& \frac{q_{1}e^{B_{1}}c}{2} -\frac{(b_{1}z+b_{0})e^{A_{2}z+B_{2}}}{2i} \\ =& \frac{q_{1}e^{B_{1}}-(b_{1}z+b_{0})e^{A_{2}z+B_{2}}}{2i}, \end{aligned}$$

then $c=-i$, note that $c\neq-i$, a contradiction.

*Subcase* 3.2, $\alpha_{1}(z)=A_{1}z+B_{1}$ and $\alpha_{2}(z)\equiv B_{2}$, similarly to the proof of Subcase 3.1, (1.6) admits a transcendental entire solution, if and only if $\frac{1}{A_{1}+i}\neq c$ (=*i*), $Q_{1}(z)\equiv q_{1}$ and $Q_{2}(z)\equiv q_{2}$. By (1.6), (4.1) and (4.2), we get

$$f'(z)=\frac{q_{1}e^{A_{1}z+B_{1}}+q_{2}e^{B_{2}}}{2} $$

and

$$f(z)=\frac{q_{1}e^{A_{1}z+B_{1}}}{2A_{1}}+\frac{q_{2}e^{B_{2}}z}{2}+c_{6},\quad e^{A_{1}c}-1=-iA_{1}, c=i, $$

where $B_{1}$, $B_{2}$, $c_{6}$ are constants and $q_{1}$, $q_{2}$ are non-zero constants.

*Subcase* 3.3, $\alpha_{1}(z)=A_{1}z+B_{1}$ and $\alpha_{2}(z)=A_{2}z+B_{2}$, by (4.6) and (4.7), we obtain, respectively,

4.8 $$ Q_{1}(z+c)-Q_{1}(z)\equiv\frac{1}{A_{1}+i}Q_{1}'(z) \quad \mbox{and}\quad A_{1}+i=ie^{A_{1}c} $$

and

4.9 $$ Q_{2}(z+c)-Q_{2}(z)\equiv\frac{1}{A_{2}-i}Q_{2}'(z) \quad \mbox{and}\quad A_{2}-i=-ie^{A_{2}c}. $$

Clearly, $A_{1}\neq A_{2}$. In what follows, we discuss four subcases: Subcase 3.3.1, $\frac{1}{A_{1}+i}\neq c$ and $\frac{1}{A_{2}-i}\neq c$; Subcase 3.3.2, $\frac{1}{A_{1}+i}=c$ and $\frac{1}{A_{2}-i}\neq c$; Subcase 3.3.3, $\frac{1}{A_{1}+i}\neq c$ and $\frac{1}{A_{2}-i}=c$; Subcase 3.3.4, $\frac{1}{A_{1}+i}=\frac{1}{A_{2}-i}=c$.

*Subcase* 3.3.1, $\frac{1}{A_{1}+i}\neq c$ and $\frac{1}{A_{2}-i}\neq c$, by (4.8), (4.9) and Lemma 2.4, we have $Q_{1}(z)\equiv q_{1}$ and $Q_{2}(z)\equiv q_{2}$, where $q_{1}$ and $q_{2}$ are non-zero constants. By (1.6), (4.1) and (4.2), we get

$$f'(z)=\frac{q_{1}e^{A_{1}z+B_{1}}+q_{2}e^{A_{2}z+B_{2}}}{2} $$

and

$$f(z)=\frac{q_{1}e^{A_{1}z+B_{1}}}{2A_{1}} +\frac{q_{2}e^{A_{2}z+B_{2}}}{2A_{2}}+c_{7}. $$

*Subcase* 3.3.2, $\frac{1}{A_{1}+i}=c$ and $\frac{1}{A_{2}-i}\neq c$. By $\frac{1}{A_{1}+i}=c$, (4.8) and Lemma 2.4, we have $Q_{1}(z)\equiv q_{1}$ or $Q_{1}(z)=a_{1}z+a_{0}$, where $a_{1}$, $q_{1}$ are non-zero constants and $a_{0}$ is a constant. By $\frac{1}{A_{2}-i}\neq c$, (4.9) and Lemma 2.4, we have $Q_{2}(z)\equiv q_{2}$, where $q_{2}$ is a non-zero constant. If $Q_{1}(z)\equiv q_{1}$ and $Q_{2}(z)\equiv q_{2}$, the same as Subcase 3.3.1. If $Q_{1}(z)=a_{1}z+a_{0}$ and $Q_{2}(z)\equiv q_{2}$, by (1.6), (4.1) and (4.2), we get

$$f'(z)=\frac{(a_{1}z+a_{0})e^{A_{1}z+B_{1}}+q_{2}e^{A_{2}z+B_{2}}}{2} $$

and

$$f(z)=\frac{ (a_{1}z+a_{0}-\frac{a_{1}}{A_{1}} )e^{A_{1}z+B_{1}}}{2A_{1}} +\frac{q_{2}e^{A_{2}z+B_{2}}}{2A_{2}}+c_{8}. $$

*Subcase* 3.3.3, $\frac{1}{A_{1}+i}\neq c$ and $\frac{1}{A_{2}-i}=c$, similarly to the proof of Subcase 3.3.2, we can obtain

$$f(z)=\frac{q_{1}e^{A_{1}z+B_{1}}}{2A_{1}} +\frac{q_{2}e^{A_{2}z+B_{2}}}{2A_{2}}+c_{7}, $$

or

$$f(z)=\frac{q_{1}e^{A_{1}z+B_{1}}}{2A_{1}} +\frac{ (b_{1}z+b_{0}-\frac{b_{1}}{A_{2}} )e^{A_{2}z+B_{2}}}{2A_{2}} +c_{9}, $$

where $B_{1}$, $B_{2}$, $b_{0}$, $c_{7}$, $c_{9}$ are constants and $A_{1}$, $A_{2}$, $q_{1}$, $q_{2}$, $b_{1}$, *c* are non-zero constants.

*Subcase* 3.3.4, $\frac{1}{A_{1}+i}=\frac{1}{A_{2}-i}=c$. By $\frac{1}{A_{1}+i}=c$, (4.8) and Lemma 2.4, we have $Q_{1}(z)\equiv q_{1}$ or $Q_{1}(z)=a_{1}z+a_{0}$, where $a_{1}$, $q_{1}$ are non-zero constants and $a_{0}$ is a constant. By $\frac{1}{A_{2}-i}=c$, (4.9) and Lemma 2.4, we have $Q_{2}(z)\equiv q_{2}$ or $Q_{2}(z)=b_{1}z+b_{0}$, where $b_{1}$, $q_{2}$ are non-zero constants and $b_{0}$ is a constant. If $Q_{1}(z)\equiv q_{1}$ and $Q_{2}(z)\equiv q_{2}$, the same as Subcase 3.3.1. If $Q_{1}(z)=a_{1}z+a_{0}$ and $Q_{2}(z)\equiv q_{2}$, the same as Subcase 3.3.2. If $Q_{1}(z)\equiv q_{1}$ and $Q_{2}(z)=b_{1}z+b_{0}$, the same as Subcase 3.3.3. If $Q_{1}(z)=a_{1}z+a_{0}$ and $Q_{2}(z)=b_{1}z+b_{0}$, by (1.6), (4.1) and (4.2), we get

$$f'(z)=\frac{(a_{1}z+a_{0})e^{A_{1}z+B_{1}}+(b_{1}z+b_{0})e^{A_{2}z+B_{2}}}{2} $$

and

$$f(z)=\frac{ (a_{1}z+a_{0}-\frac{a_{1}}{A_{1}} )e^{A_{1}z+B_{1}}}{2A_{1}} +\frac{ (b_{1}z+b_{0}-\frac{b_{1}}{A_{2}} )e^{A_{2}z+B_{2}}}{2A_{2}}+c_{10}. $$

*Case* 4, $e^{\alpha_{1}(z)-\alpha_{1}(z+c)}$, $e^{\alpha_{2}(z)-\alpha_{1}(z+c)}$ and $e^{\alpha_{2}(z+c)-\alpha_{1}(z+c)}$ are all constants, that is, $\alpha_{1}(z)-\alpha_{1}(z+c)$, $\alpha_{2}(z)-\alpha_{1}(z+c)$ and $\alpha_{2}(z+c)-\alpha_{1}(z+c)$ are all constants. Note that $\alpha_{1}(z)$ and $\alpha_{2}(z)$ are not constants simultaneously, then $\alpha_{1}(z)=Az+B_{1}$, $\alpha_{2}(z)=Az+B_{2}$ and $\alpha(z)=2Az+D$, where *A* is non-zero constant and $B_{1}$, $B_{2}$, *D* ($=B_{1}+B_{2}$) are constants. Therefore, by (1.6), (4.1) and (4.2), we have $f(z)=B(z)e^{Az}+c_{0}$, where $B(z)$ satisfies $[B'(z)+AB(z)]^{2}+[B(z+c)e^{Ac}-B(z)]^{2}=Q(z)e^{D}$.

This completes the proof of Theorem 1.2.

## Proof of Theorem 1.3

Suppose that $f(z)$ is a transcendental entire function of finite order satisfying (1.9). In what follows, we will discuss four cases: Case 1, $m=n\geq2$; Case 2, $m>n$; Case 3, $n>m\geq2$; Case 4, $n\geq3$, $m=1$.

*Case* 1, $m=n\geq2$. If $m=n=2$, note that $P(z)$ and $Q(z)$ are non-constant polynomials, by Theorem *D*, we see that (1.9) has no transcendental entire solutions of finite order. If $m=n\geq3$, rewriting (1.9) as $\frac{1}{Q(z)}(f'(z))^{n}+\frac{P(z)}{Q(z)}f^{m}(z+c)=1$, by Theorem *E*, we see that (1.9) has no transcendental entire solutions of finite order.

*Case* 2 and *Case* 3, similarly to the proof of the Case 1 and Case 2 of [10], Theorem 1.2, we can also obtain (1.9) has no transcendental entire solutions of finite order.

*Case* 4, $n\geq3$, $m=1$. Differentiating (1.9), we get

$$n\bigl(f'(z)\bigr)^{n-1}f''(z)+P'(z)f(z+c)+P(z)f'(z+c)=Q'(z). $$

Substituting (1.9) into the above equation yields

5.1 $$ \bigl(f'(z)\bigr)^{n-1} \biggl[nf''(z)- \frac{P'(z)}{P(z)}f'(z) \biggr] =-P(z)f'(z+c)+Q'(z)-Q(z) \frac{P'(z)}{P(z)}. $$

Denote $F(z)=f'(z)$, $\varphi(z)=nf''(z)-\frac{P'(z)}{P(z)}f'(z)=nF'(z)-\frac{P'(z)}{P(z)}F(z)$. Then (5.1) can be rewritten as

5.2 $$ F^{n-1}(z)\varphi(z)=-P(z)\frac{F(z+c)}{F(z)}F(z)+Q'(z)-Q(z) \frac {P'(z)}{P(z)}. $$

By Lemma 2.5, we see that $m (r,\frac{F(z+c)}{F(z)} )=S(r,F)$ and $m (r,Q'(z)-Q(z)\frac{P'(z)}{P(z)} )=S(r,F)$, note that $n-1\geq2$, by Lemma 2.1, we have

$$m\bigl(r,\varphi(z)\bigr)=S(r,F)\quad \mbox{and} \quad m\bigl(r,F(z)\varphi(z) \bigr)=S(r,F). $$

We see that $\varphi(z)\not\equiv0$, otherwise $(f'(z))^{n}=F^{n}(z)\equiv AP(z)$, where *A* is a non-zero constant, a contradiction. Note that $f(z)$ is a transcendental entire function, then $N(r,\varphi(z))=S(r,F)$ and

$$\begin{aligned} T\bigl(r,F(z)\bigr)=m\bigl(r,F(z)\bigr) \leq& m\bigl(r,F(z)\varphi(z)\bigr)+m \biggl(r,\frac{1}{\varphi (z)} \biggr) \\ \leq& m\bigl(r,\varphi(z)\bigr)+N\bigl(r,\varphi(z)\bigr)+S(r,F)=S(r,F), \end{aligned}$$

that is, $T(r,f'(z))=T(r,F(z))\leq S(r,F)=S(r,f')$, a contradiction.

This completes the proof of Theorem 1.3.
